# Effect of GO on the Structure and Properties of PEG/Biochar Phase Change Composites

**DOI:** 10.3390/polym15040963

**Published:** 2023-02-15

**Authors:** Weijie Chen, BingBing Zhang, Sheng Wang, Bin Xue, ShiWang Liu, MingZhe An, Zhao Yang, Guomin Xu

**Affiliations:** 1 National Engineering Research Center for Compounding and Modification of Polymer Materials, Guiyang 550014, China; 2 College of Resources and Environmental Engineering, Guizhou University, Guiyang 550025, China; 3 Department of Polymer Material and Engineering, College of Materials and Metallurgy, Guizhou University, Guiyang 550025, China

**Keywords:** phase change materials (PCMs), graphene oxide (GO), biochar, surfactants, thermal conductivity, the photothermal conversion efficiency

## Abstract

In recent years, phase change materials (PCMs) have been widely used in waste heat utilization, buildings, and solar and wind energy, but with a huge limitation from the low thermal conductivity, photothermal conversion efficiency, and low latent heat. Organic PCMs are eyecatching because of its high latent heat storage capability and reliability, but they still suffer from a lack of photothermal conversion and sharp stability. Here, we prepared sharp-stable PCMs by establishing a carbon material frame system consisting of graphene oxide (GO) and biochar. In particular, surfactants (CTAB, KH-560 and KH-570) were employed to improve the dispersity of GO in PEG. The differential scanning calorimetry results shows that the latent heat of PEG modified by CTAB grafted GO (PGO-CTAB) was the highest (191.36 J/g) and increased by 18.31% compared to that of pure PEG (161.74 J/g). After encapsulation of PGO-CTAB in biochar, the obtained composite PCM with the amount of biochar and PGO-CTAB in weight ratio 4:6 (PGO-CTAB/CS6(6)) possesses relatively high latent heat 106.51 J/g with good leak resistance and thermal stability, and with obviously enhanced thermal conductivity (0.337 W/(m·K)) and photothermal conversion efficiency (77.43%), which were higher than that of PEG6000 (0.325 W/(m·K), 44.63%). The enhancement mechanism of heat transfer and photothermal conversion on the composite PCM is discussed.

## 1. Introduction

In today’s rapidly developing economy and society, methods for energy conversion and storage have become a focus of attention [1]. Phase change materials (PCMs) have been considered an efficient strategy to relieve the pressure of the global energy crisis since they can significantly improve energy storage efficiency due to their excellent performance on chemical stability and various thermal properties [2]. However, the low thermal conductivity, photothermal conversion efficiency, and low latent heat limit the application of PCMs. Therefore, improving the thermal performance of PCMs has been the research focus [3].

Organic PCMs are eyecatching because of its high latent heat storage capability, thermal stability, and reliability. However, the problem of leakage during phase transition and the lack of photothermal conversion ability remains a major obstacle to their practical application and development [4]. To balance the latent heat, leak resistance, thermal stability, thermal conductivity, and photothermal conversion ability of organic PCMs, a variety of composite methods were studied.

One approach is to disperse highly thermally conductive particles (e.g., graphite powder, carbon nanotube/graphene nanosheets, and micro/nanometallic particles) into organic PCMs, which improves the thermal properties of the material [5,6,7,8]. Kant et al. found that introducing ultra-low density multi-nanoporous 3D ultra-lightweight graphene nanoparticles into PCMs could enhance their thermal performance and obtain high adsorption capacity to organic liquids [9]. A similar study used different volume ratios (1%, 3%, and 5%) of graphene nanosheets mixed into organic, inorganic, and paraffinic PCMs. The small volume ratios of graphene nanosheets significantly improved the effective thermal conductivity of the latent heat storage media. However, this method failed to gain the thermal stability and reliability of PCMs. After several melting and solidification cycles, aggregation or precipitation of these dispersed particles occured, which significantly affects the material’s thermal properties [10]. Maintaining good dispersity of added particles during multiple freeze−thaw cycles seems key for thermal stability and reliability of the composite PCMs. Graphene oxide (GO) has oxygen-containing functional groups on its surface [11]. With effective surface modification, its dispersion properties in organic PCMs could be improved [12]. For example, to avoid the severe agglomeration problem of added GO, Cao et al. employed the polynorbornene and octadecylamine functionalized graphene oxide nanosheets (C18-rGO) dispersing into paraffinic PCMs, which than effectively enhance the structural stability of the phase change material [13]. The dispersion of GO in PEG is closely related to its degree of oxidation [14]. It was found that the modification of GO using surface modifiers resulted in better binding to PEG and improved its dispersion properties as well as its crystal structure [15,16,17].

Another approach is to introduce porous materials of highly conductive and thermally conductive structures as a support matrix for liquid organics. With lamellar, honeycomb, or foam structures, the usual support matrix includes mesoporous silica, montmorillonite nanosheets, activated carbons, and biochar [18]. The composite PCMs showed good thermal stability and chemical stability. As a response to the call for fabricating highly value-added supporting materials, Biochar could be employed as the adsorbent for preventing leakage of organic PCMs. It has been well-known as an inexpensive (up to six times cheaper than commercial activated carbons), sustainable precursor and attracted interest from broad scientific disciplines [19]. Biochars have been introduced for the adsorption of organic PCMs [20]. However, the organic PCMs encapsulated in biochar would sacrifice their latent heat storage and thermal conductivity. A reasonable design of carbon material frame system as a support matix for organic PCMs would be necessary to gain the balance the latent heat, leakage resistant, and thermal performance of organic PCMs.

In order to realize efficient solar energy storage and conversion, simple and fast PCMs production methods are required [21]. The GO and biochar materials added to phase change materials together have become a hot research topic. Biochar is directly derived from the nature of the biomass, allowing the pore structure to be adjusted to optimize thermal energy storage capacity. In our previous work, the optimal preparation condition of corn straw biochar for PEG/biochar composite PCMs with acceptable heat storage ability and good leak resistance have been studied, but the photothermal conversion still needs to be improved.

This work established a carbon material frame system consisting of graphene oxide (GO) and biochar as a support matrix for organic PCMs. In particular, three surfactants (KH560, KH570, and CTAB) were grafted on the surface of GO particles, respectively, to obtain different GO-modifiers. Among these three, the γ-methacryloxypropyltrimethoxysilane (KH-570) and γ-glycidyl ether oxypropyltrimethoxysilane (KH-560) are often used as hydrophobic modifiers on the surface of graphene oxide, which can effectively improve the dispersion of graphene oxide in the PEG system [22]. The cetyltrimethylammonium bromide (CTAB) can also improve the hydrophobicity and dispersibility of GO because of its similar polarity to the PEG molecular chain caused by its hydrophilic polar head group and hydrophobic alkyl tail. The GO-modifiers were dispersed in PEG6000 to obtain a GO-PEG PCMs, which were then loaded onto the surface and in the micropores of biochar. The effects of different surfactants on the structure and properties of GO-PEG PCMs and the enhancement mechanism of heat transfer and photothermal conversion on the GO-PEG/biochar composite PCMs were investigated.

## 2. Materials and Methods

### 2.1. Materials 

The primary raw materials used for sample preparation in this study were corn straw, polyethylene glycol with an average molecular weight 6000 g/mol (PEG6000, purity ≥ 99.0%) and graphene oxide (GO, 8.0 mg/mL, purity ≥ 99.0%). The corn straw was obtained from Guiyang City, Guizhou Province, China. PEG6000 was procured from Shanghai Alighting Biochemical Technology Co. LTD, as well as cetyltrimethylammonium bromide (CTAB, purity ≥ 99.0%),γ-(2,3-epoxypropoxy)propytrimethoxysilane (KH-560, purity ≥ 99.0%) and 3-(trimethoxysilyl)propyl methacrylate (KH-570, purity ≥ 98.0%), and GO from Shanghai Aladdin Biochemical Technology Co. Chemicals used in the experiments were all analytical reagents without any further purification.

### 2.2. Methods

#### 2.2.1. GO-Modifiers

**Figure 1:**
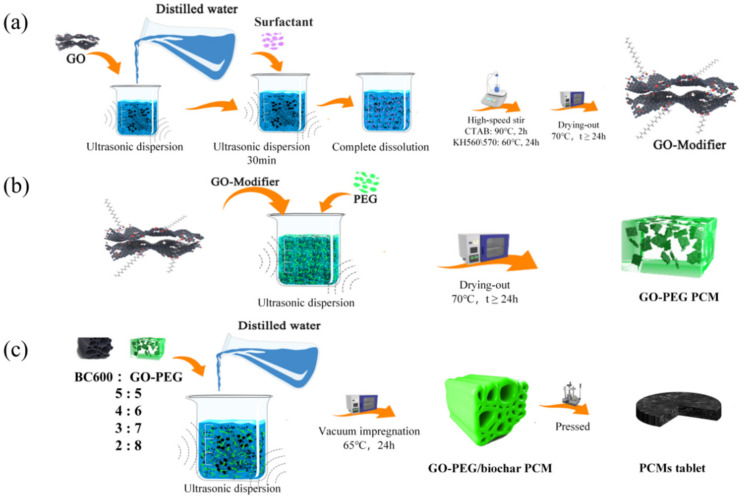
Schematic diagram of the preparation process for (**a**) GO-modifier and (**b**) GO-PEG PCMs (**c**) GO-PEG/biochar PCMs.

The schematic diagram of the preparation process for GO-modifiers is shown in Figure 1a GO with the concentration of 8.5 mg/mL was ultrasonically dispersed in 25 mL of deionized water, and CTAB (10 wt.% of GO), as a cationic surfactant, was. With a 30 min ultrasonic dispersion, CTAB was completely dissolved. The surface activation of GO occured during 2 h of high-speed stirring at 90 °C. Afterward, the above mixture was dried at 70 °C and cooled down spontaneously. The obtained GO-modifier was denoted as GO-CTAB. The similar GO activation by two other surfactant coupling agents, KH-560 and KH-570, proceeded with different stirring duration (24 h) at different working temperatures (60 °C). The corresponding GO modifiers were denoted as GO-KH560 and GO-KH570, respectively.

#### 2.2.2. GO-PEG PCMs

As shown in Figure 1b, 0.5 g PEG6000 was ultrasonically dispersed in 25 mL of deionized water with 0.6 wt.% (of PEG6000) GO, GO-CTAB, GO-KH560, or GO-KH570. A series of GO-doped PEGs were obtained after 30 min of ultrasonic dispersion at room temperature and drying at 70 °C. The resulting GO-PEG PCMs were denoted as PGO, PGO-CTAB, PGO-KH560, and PGO-KH570, respectively.

#### 2.2.3. GO-PEG/Biochar PCMs

The dried corn straw was crushed and heated to 600 °C (with a holding time of 2.5 h) in an anoxic atmosphere (with a nitrogen flow rate of 60 mL/min) and at a heating rate of 8 °C/min. After cooling spontaneously, the obtained biochar was grounded, sieved, adequately saved, and denoted as CS6.

The GO-PEG/biochar PCMs have been prepared by vacuum impregnation with compounded GO-PEGs and porous biochars. As shown in Figure 1c, 10g precursor of CS6 and PGO-CTAB in a different ratio (5:5, 4:6, 3:7, 2:8) were ultrasonically dispersed in 25 mL of deionized water for 90 min. Afterward, the precursors were dried at 65 °C in a vacuum oven for 24 h and cooled down spontaneously to obtain the GO-PEG/biochar composite PCMs, which were denoted as PGO-CTAB/CS6(5), PGO-CTAB/CS6(6), PGO-CTAB/CS6(7), and PGO-CTAB/CS6(8), respectively. The numbers (5, 6, 7, 8) in the parentheses indicate the weight ratio of PGO-CTAB towards CS6.

Specifically, a sample of PEG6000/biochar PCMs (without any GOs) in ratio of 6:4 was prepared for the purpose of comparison, and denoted as PEG6000/CS6(6). All PCMs were eventually pressed into 2 mm thick and 12.7 mm diameter tablets.

### 2.3. Material Characterizations

Fourier-transform infrared spectroscopy (FTIR: Nicolet NEXUS670, USA) was used to identify variations in the total composition of pristine GO and grafted GO-modifiers, purePEG6000 and PEG composites, through the determination of changes in functional groups on their surface. All samples were in a solid state and pressed into pellets with potassium bromide (KBr) as carriers. The light was in the wavenumber range of 4000–400 cm^−1^.

The thermal stability of surfactants (CTAB, KH570, and KH560), GO, GO-modifiers, pure PEG6000, and PEG composite PCMs were analyzed with a thermogravimetric analyzer (TGA: TA Q50, America). All samples were heated to 600 °C at a heating rate of 10 °C min^–1^ under a nitrogen atmosphere.

The differential scanning calorimetry (DSC: TA Q10, America) was employed to study the heating-cooling behavior of PCMs. The heating and cooling procedure were conducted in the range of 0 °C–90 °C and 90 °C–0 °C, respectively, at a heating (cooling) rate of 5 °C min^–1^ under a nitrogen atmosphere. The thermal cycle stability of the sample was tested under 100 heating-cooling cycles.

The leakage resistance and shape stability of PCMs were recorded at 70 °C in the constant temperature oven in 0, 60, and 180 min, respectively. It is worth noting that 70 °C was a high melting temperature compared to the pure PEG6000.

The crystalline structure of PCMs was studied by the X-ray diffractometer (XRD, X’Pert PRO). The rate of scanning and range was 10 min^–1^ and 10–80, respectively.

The thermal conductivity and photothermal conversion of pure PEG6000, PEG6000/biochar, and GO-PEG/biochar PCM samples were studied by the laser flash method (NETZSCH LFA 467 HyperFlash, 25 °C) with the light intensity of 100 mW cm and recording interval of 1 s.

## 3. Results and Discussion

### 3.1. Microstructure and Thermal Stability of GO-Modifiers

**Figure 2:**
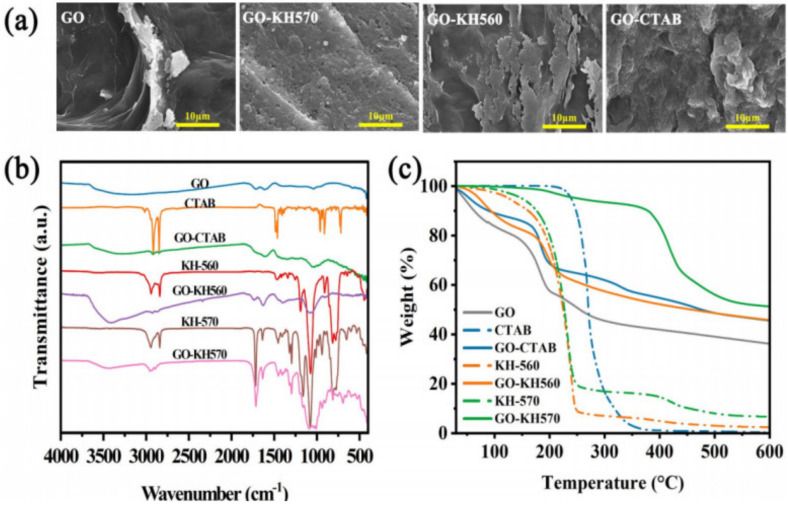
(**a**) SEM images of GO-modifiers, (**b**) FTIR, and (**c**) TGA curves of GO, surfactants, and corresponding GO-modifiers.

The surface morphology, composition, and thermostability of various GO-modifiers were studied, including the pristine GO and GO-modifiers surface-activated by CTAB, KH-560, and KH-570. Figure 2a shows the SEM images of all GO-modifiers. The pristine GO consists of wrinkled layers of smooth carbon sheets. In turn, the images of GO-CTAB, GO-KH560, and GO-KH570 show a preserved lamellar structure from GO with rough surfaces caused by different surfactants. Compared with GO-KH560 and GO-KH570, the GO-CTAB exhibits an irregular morphology with a more homogeneous texture may lead to its higher dispersibility than others in further PEG compounds.

In the FTIR spectrum of Figure 2b, GO has a broad O–H stretching vibration band at 3221 cm^−^^1^, carboxyl C=O stretching band at 1718 cm^−^^1^, and C–O stretching vibration at 1038 cm^−^^1^. As for the FTIR spectrum of pure CTAB, characteristic peaks at 2917 cm^−^^1^, 2849 cm^−^^1^, 1473 cm^−^^1^, 961 cm^−^^1^, 911 cm^−^^1^, and 719 cm^−^^1^ corresponded to vibrational bands of CH (symmetric and asymmetric stretching vibrations), CH_2_, N(CH_3_)_2_, and CH functional group, were identified. The vibration peaks of GO-CTAB combines the majority of above peaks of both GO and CTAB, confirming a successful grafting on the surface of this GO-modifier. Similarly, the effective combination of GO and other respective surfactant coupling agents, KH-560 and KH-570, can be observed [23].

The thermal stability of the GO-modifiers was evaluated by TG analysis. Figure 2c shows the resulting TG curves of the pristine GO, all three surfactants, and their corresponding GO-modifiers. The TGA curves show different decomposition patterns based on the type of samples. As for the surfactants, the thermal decomposition of KH-560 and KH-570 takes place at two major steps with an onset temperature of around 100 °C, while the deterioration of CATB is just one step with a higher onset temperature of around 250 °C. All three surfactants were more stable than the pristine GO. With the respective combination of CTAB, KH-560, and KH-570, the thermal stability of GO-CTAB, GO-KH560, and GO-KH570 were enhanced. Among them, GO-KH570 shows the most enhanced stability with the highest onset temperature (400 °C) of its second decomposition region.

### 3.2. Microstructure and Thermal Property of GO-PEG PCMs

**Figure 3:**
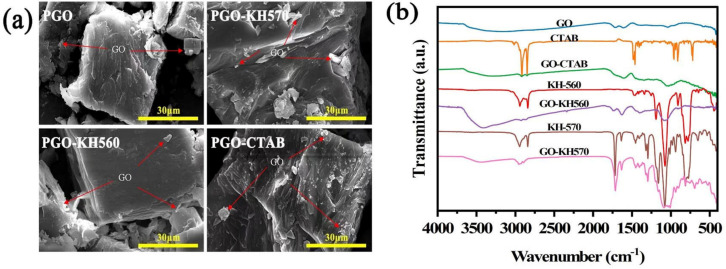
(**a**) SEM images of GO-PEG PCMs, (**b**) FTIR of GO, PEG6000 and GO-PEG PCMs.

Small differences in surface morphology can be observed between PGO and other GO-PEG PCMs, as Figure 3a shows relatively smooth surfaces with cracks and fragments. But it seems that more fragments can be found on the surface of solid-state PGO-CTAB, implying the significant effect of CTAB on the crystal aggregation of corresponding GO-PEG PCMs.

In Figure 3b, the O–H and CH_2_ stretching vibration band at 3423 cm^−^^1^ and 2882 cm^−^^1^, CH bending vibration band at 1467 cm^−^^1^ and 1360 cm^−^^1^, C–O–C symmetric stretching vibrations at 1104 cm^−^^1^ on the FTIR spectrum of PEG6000 can be obviously seen. All other GO-PEG PCMs (including PGO, PGO-CTAB, PGO-KH560, and PGO-KH570) show the same vibration regions of pure PEG6000 and the carboxyl C=O stretching band at 1718 cm^−^^1^ from GO. However, the C=C stretching band at 1639 cm^−^^1^ on the spectrum of GO-PEG PCMs is weaker than that of pure PEG6000, implying weak interface bonds between PEG6000 and the GO-modifiers [24].

**Figure 4:**
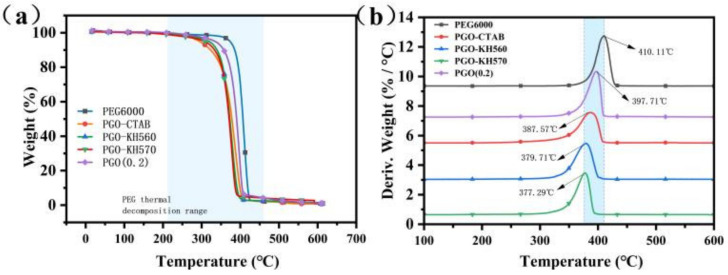

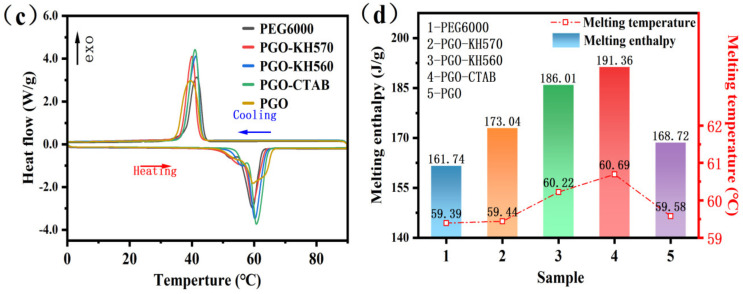
(**a**) TGA, (**b**) DTG, (**c**) DSC curves, (**d**) phase transition enthalpy and temperature of PEG6000 and GO-PEG PCMs.

The thermal stability of the GO-PEG PCMs was studied by TGA. Figure 4a,b shows the TGA and DTG curves of PEG6000, PGO, PGO-CTAB, PGO-KH560, and PGO-KH570. The one-stage thermal decomposition patterns on all curves were similar, while the decomposition of GO-PEG PCMs occurs at a lower temperature region than that of pure PEG6000, as a result of the high performance on thermal conductivity of GO-modifiers and the constraint effect from dispersed GO-modifiers limiting the normal motion of PCM during phase transition. With much more functional groups on the surface of GO-modifiers as shown in Figure 4b, the surfactants grafted on the GO surface have improved the dispersibility of GO in PEG6000.

The phase change behaviors of PEG6000 and GO-PEG PCMs, including the phase change temperatures and latent heat, were evaluated using DSC. The DSC curves of PEG6000 and GO-PEG PCMs are shown in Figure 4c, and the calculated test data are summarized in Table 1. As shown in Figure 4d, the increased trend of the melting temperature as well as the latent heat of pure PEG6000, PGO, PGO-CTAB, PGO-KH560, and PGO-KH570 can be found. The latent heat of PGO-CTAB, PGO-KH560, and PGO-KH570 (173.04, 186.01, 191.36 J/g, respectively) are higher than that of PGO (168.72 J/g), demonstrating the improved crystallization behavior of PEG6000 on nucleation sites contributed from surface grafted GO-modifier. The surfactants have improved both dispersibility and compatibility of GOs in PEG6000. Among all GO-PEG PCMs, the latent heat of PGO-CTAB was the highest and increased by 18.31% compared to that of PEG6000 (161.74 J/g), so PGO-CTAB was considered as the best thermal enhancement GO-modifier and the best candidate for the further preparation of biochar composite PCMs. PGO-CTAB has a better latent heat of phase change (77.27–106.5 J/g) compared to porous diatomaceous earth powder with adsorbed polyethylene glycol (PEG) PCMs [25].

### 3.3. Microstructure and Thermal Performance of GO-PEG/Biochar PCMs

**Figure 5:**
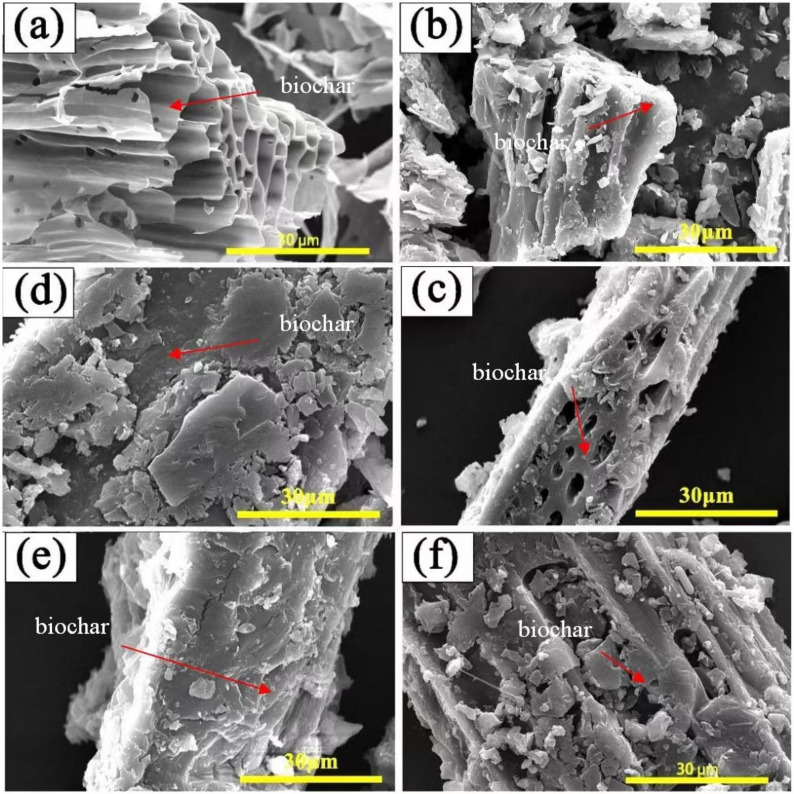
SEM images of (**a**) CS6, (**b**) PGO-CTAB/CS6(5), (**c**) PGO-CTAB/CS6(6), (**d**) PGO-CTAB/CS6(7), (**e**) PGO-CTAB/CS6(8) and (**f**) PEG6000/CS6(6).

Figure 5a shows the SEM photographs of the used biochar, BC600, under a pyrolysis temperature of 600 °C. It is seen that BC600 shows a honeycomb porous structure, with its carbonaceous macropores as channels for adsorbates entering the porous system locating on the wall [26]. The micromorphology of various GO-PEG/biochar PCMs with a different weight ratio of PGO-CTAB to CS6 was given. As shown in Figure 5b–e, PGO-CTAB was totally introduced into the surface and pore of biochar CS6. When the ratios of PGO-CTAB: CS6 were 5:5 and 6:4, the macropores on the biochars had not been filled completely yet, indicating the efficient adsorption capacity of the porous carbon materials to PGO-CTAB. Compared with the surface morphology of PGO-CTAB/CS6(6) and PEG6000/CS6(6), as shown in Figure 5c,e, respectively, the GO-CTAB modified PEG6000 tended to enter into the channels through the carbonaceous macropores on biochars. The addition of GO-CTAB enhanced the adsroption performance of PEG6000 on CS6.

**Figure 6:**
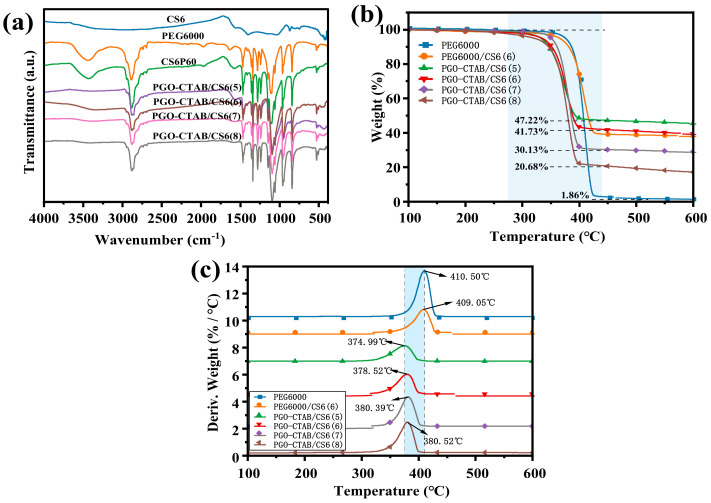
(**a**) FTIR curves, (**b**) TGA, and (**c**) DTG curves of PEG6000, PEG6000/CS6(6) and PGO-CTAB/CS6(5\6\7\8) PCMs.

Figure 6 shows the FTIR spectra, TGA, and DTG curves of PEG6000, PEG6000/CS6(6), and various GO-PEG/biochar PCMs. The FTIR spectra confirm that, with both vibration regions of CS6 and PEG6000 shown on all GO-PEG/biochar PCMs, the PGO-CTAB coupled with CS6 by physical adsorption without any interface chemical reactions between them. What is worth mentioning is that, the C=O vibration peak of GO-PEG/biochar PCMs at 1579 cm^−^^1^ has a weaker intensity than that of the PEG6000/CS6(6), and also shows the red shift (that of PEG6000/CS6(6) is at 1576 cm^−^^1^), due to the stereo-hindrance effects caused by GO-CTAB [27].

The thermal stability of the composites was studied by TGA and DTG methods. As shown in Figure 6b,c, the TGA and DTG curves of all samples demonstrate similar decomposition patterns. As for the results of PEG6000 and PEG6000/CS6(6), their onset decomposition temperature and maximum weight loss temperature are pretty close corresponding to the pyrolysis of PEG6000 chains, while these of PEG6000/CS6 PCMs are slightly lower, which are consistent with the above conclusion for GO-CTAB, that is, due to its high thermal conductivity and its constraint effect to the normal motion of PEG6000 chains. Meanwhile, the value of the maximum weight loss rate of PEG6000/CS6 PCMs declined gradually with the decreasing weight ratio of PGO-CTAB in the composite PCMs, which can be due to the presence of the limitation of the evaporation and pyrolysis of biochar compounds, consequently improving the high char residues.

**Figure 7:**
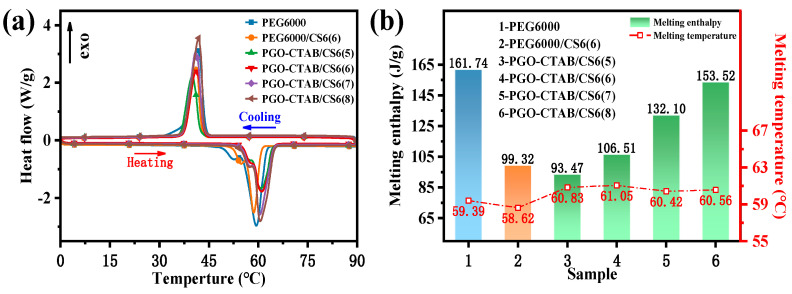
(**a**) DSC curves (**b**) and latent heat values of PEG6000, PEG6000/CS6(6) and PGO-CTAB/CS6(5\6\7\8) PCMs.

The DSC results, as shown in Figure 7a, were used to calculate the phase change temperature and the melting enthalpy of PEG6000, PEG6000/CS6(6), and PGO-CTAB/CS6 PCMs with the various weight ratio of PGO-CTAB and CS6. The calculated values are shown in both Figure 7b and Table 2. The melting curve of the PGO-CTAB/CS6(8) had a single peak at 60.56 °C with a melting enthalpy *ΔHm* of 153.52 J/g. The melting points of PGO-CTAB/CS6 remained the same as for pure PEG6000 (59.39–61.05 °C). The melting enthalpy was increased proportionally to PEG6000 content in the composites and ranged from 93.47 J/g to 153.52 J/g depending on the PGO-CTAB mass fraction. It was reported that the interaction of surface tension, weak hydrogen bonding, and capillary forces generated in the encapsulation process of PCMs caused the shift of phase change temperature of the obtained compositions [28], suggesting the effects of interaction caused by porous structure (see in Figure 5a) and surface functional groups (see in Figure 6a) of biochars towards the PGO-CTAB on its crystallization properties [29].

Since the melting and freezing enthalpies of the composites clearly depend on the mass fraction of pure PCMs (see Table 2), the loading efficiency E of PEG6000 in composite PCMs was calculated by [30]:

(1) $$E=\frac{\Delta H_{M,comp}+\Delta H_{F,comp}}{\Delta H_{M,PCM}+\Delta H_{F,PCM}}\times 100\%$$

where *∆H*_M,comp_ and *∆H*_F,comp_ are the melting and freezing enthalpies of the PGO-CTAB/CS6 composite, while *ΔH*_M,PCM_ and *ΔH*_F,PCM_ are the melting and freezing enthalpies of pure PEG6000. In the phase-change composites, the loading efficiency indicates the heat storage and release performance towards the loaded PEG6000. The calculated results of E are listed in Table 2. It is interesting to find that the E value of PEG6000/CS6(6) is 60.4%, close to its weight ratio of PEG6000 (60 wt.%), but the *E* values of all PGO-CTAB/CS6(5/6/7/8) are higher than their practical weight ratio of PEG6000 (50/60/70/80 wt.%), demonstrating the thermal enhancement of GO-CTAB in PCMs.

The *F_c_* is referred to as the crystallinity of PEG in the composites, which also means the proportion of the pure PCMs in the composites that can effectively store and release thermal energy through phase transitions, was calculated by [31]:

(2) $$F_{C}=\frac{\left(\Delta H_{M,comp}+\Delta H_{F,comp}\right)\times \Delta H_{M,PCM}}{\left(\Delta H_{M,PCM}+\Delta H_{F,PCM}\right)\times \Delta H_{M,comp}}\times 100\%$$

The calculated results are listed in Table 3. The calculated thermal storage capability of PEG6000/CS6 was 98.37%, whereas that of PGO-CTAB/CS6 PCMs was higher than 99.00%. The high crystallinity of PEG suggests that the addition of GO-CTAB helped to eliminate the confinement effects when PEG6000 was loaded on biochars in restricted volume, and PEG6000 adsorbed on the biochar surface and micropores is free to undergo reversible phase transitions [32]. Meanwhile, the crystallinity of PEG in GO-PEG PCMs are of 98.31–98.96%, which are lower than that in GO-PEG/biochar PCMs (all above 99%), indicating the induction of crystallization of biochar towards PEG6000.

**Figure 8:**
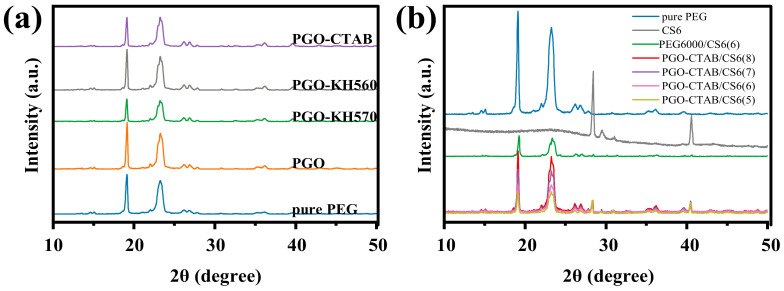
XRD curves of (**a**) pure PEG6000 and GO-PEG PCMs, (**b**) PEG6000/CS6(6) and PGO-CTAB/CS6(5\6\7\8) PCMs.

To further understand why the GO-modifiers can improve the latent heat of PEG in composite PCMs, the crystalline performance of these PCMs were studied by XRD. Figure 8 shows the XRD patterns of pure PEG6000, GO-PEG PCMs, PEG6000/CS6(6), and PGO-CTAB/CS6(5\6\7\8) PCMs, respectively. Representative peaks of PEG can be seen for both pure PEG6000 can be seen for all GO-PEG PCMs, PEG6000/CS6(6), and PGO-CTAB/CS6(5\6\7\8) PCMs, but peak strength is decreased. The pore structure and surface functional groups of biochar can affect the crystal orientation and confine the movement of PEG6000 molecular, thus affecting the crystallinity of PEG6000. The crystallinity of PEG6000 in the GO-PEG/biochar PCMs is one reason leading to the deviation of DSC curves of the composite PCMs compared to pure PEG6000 [33], as seen in Figure 4c and Figure 7a.

The crystallization properties of PEG6000 in PCMs can be evaluated by calculating its average crystal size via Scherrer formula (Equation (3)) [34] and the calculated values are listed in Table 4.

(3) $$D=\frac{K\lambda }{\beta \cos\left(\theta \right)}$$

where *K* is a constant, λ is the X-ray wave length, *β* was full width at half maximum (FWHM), and *θ* is the Bragg reflection angle.

The average crystal size of PEG6000 in all composites was 11.17–11.83 nm, smaller than that of pure PEG6000 (12.04 nm). As a result of that, the different surface grafting groups on GO offered by these surfactants play as nucleation points, and the crystal grain of PEG modified by GO under surfactant treatments are smaller than that of that of PEG with pristine GO. When the GO-PEG PCMs was encapsulated in biochar, more nucleation points has formed on the large specific external surface of biochar. Thus, the PEG grains grow smaller.

**Figure 9:**
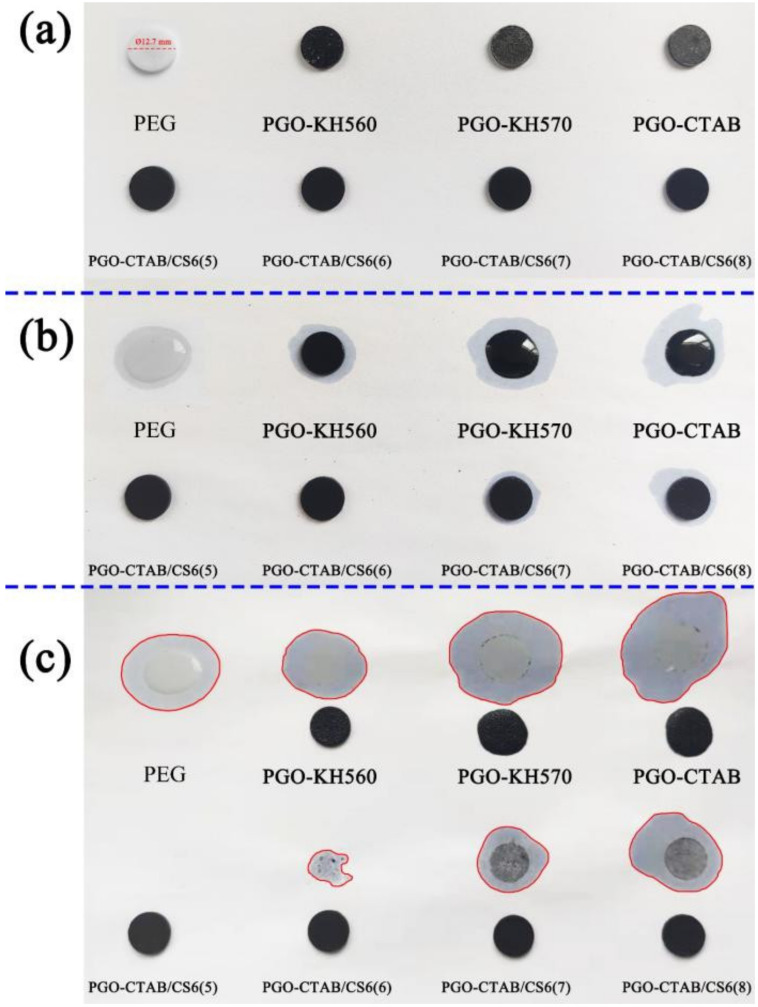
Leakage stability diagram of various PCMs at (**a**) 0 min, (**b**) 60 min, and (**c**) 180 min.

The leakage resistant of GO-PEG PCMs and PGO-CTAB/CS6(5\6\7\8) PCMs were studied by in-situ recording their states at different time under continuous heating at 70 °C, as seen in Figure 9. KH560 and PGO-CTAB) were gradually melted as test time goes on and almost wholly melted at 1800 s. However, with the encapsulation in biochars, PGO-CTAB/CS6 PCMs perform better leakage resistance with excellent shape-stability, which was mainly due to the capillarity, strong surface tension, and hydrogen bond interaction of the microstructure of biochars towards PEG. Among them, PGO-CTAB/CS6(5) shows the best leak resistance [35].

The leakage area of these PCMs was measured using the Image J analysis program, and the leakage rate Φ was calculated according to Equation (4) and listed in Table 5.

(4) $$\begin{array}{c} \Phi \;=\frac{A_{\mathrm{PCMs}}}{A_{\mathrm{PEG}}} \end{array}$$

where *A_PCMs_* and *A_PEG_* are the leakage area of PCMs and pure 6000, respectively. The result demonstrates that the addition of modified GO and biochar significantly improved the shape stability and leakage resistance of PEG [36,37].

### 3.4. Thermal Conductivity and Photo-Thermal Conversion Performance of GO-PEG/Biochar PCMs

The ability of the materials to conduct heat energy can be valued by their thermal conductivity (*K*), which can be calculated from Equation (5), as follows:

*K* = *α* × *ρ* × *C_p_* (5)

where *K* is the thermal conductivity, *ρ* is the density, and *C_p_* is the specific heat of the prepared sample.

**Figure 10:**
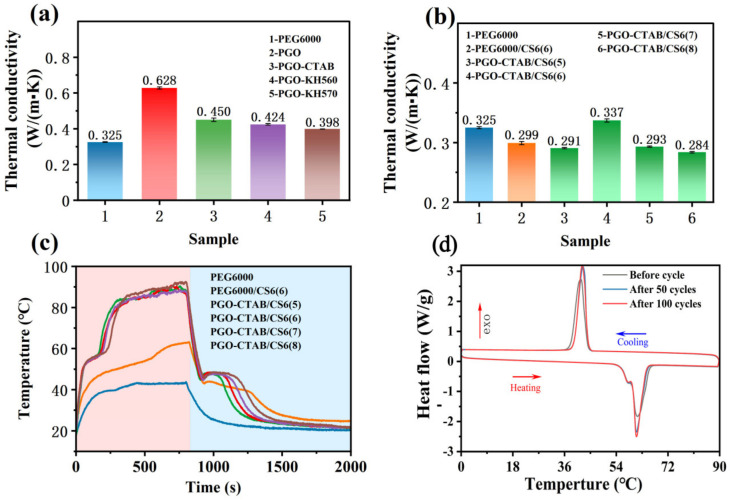
(**a**,**b**) Thermal conductivity at 25 °C and (**c**) photo-thermal conversion curves of various PCMs. (**d**) Thermal cycle stability of PGO-CTAB/CS6(6) phase change composites.

Figure 10 shows the thermal conductivity of pure PEG6000, GO-PEG PCMs, PEG6000/CS6(6), and PGO-CTAB/CS6(5\6\7\8) PCMs at 25 °C. The relevant parameters of the thermal conductivity are recorded in Table 6. Figure 10a shows that the thermal conductivity of PGO is 0.628 W/(m·K), indicating the addition of GO (0.6 wt.% of PEG6000) improves the thermal conductivity of PEG by 93.5%, which is due to the thermal conductivity frame offered by dispersive GO particles and its induced crystallization effects towards PEG [38]. Thus, in this case, GO can be regarded as the thermally enhanced particle for PCMs.

Meanwhile, the thermal conductivity of the GO-PEG PCMs treated by surfactants is slightly reduced compared with that of PGO, implying the negative influence of these surfactants on the GO induced crystallization effects toward PEG. Among them, the thermal conductivity of PGO-CTAB/CS6(6) (0.337 W/(m·K)) is of practical significance, a little higher than that of PEG6000. As far as the PGO-CTAB/CS6(5\7\8) is concerned, their thermal conductivity is lower than that of pure PEG and PGO-CTAB because the encapsulation of PCMs into biochar may leave air gaps and residual gas inside the composite leading to breaks of its thermal conductivity pathway. The thermal stability and reliability of PGO-CTAB/CS6(6) was confirmed by the freeze−thaw cycling test100 times. As shown in Figure 10d, the pattern of characteristic peaks on the after-100-cycles curve almost remained, and the calculated enthalpy capacity was 98.57% as the initial value, implying the excellent stability of PGO-CTAB/CS6(6) [39].

The photo-thermal conversion of pure PEG6000, PEG6000/CS6(6), and PGO-CTAB/CS6(5\6\7\8) PCMs was studied by using an infrared radiation source (200 Mw/cm^2^). The infrared radiation can drive the energy conversion and energy storage of PCMs. With infrared radiation, the temperature of samples was raised, and the corresponding time-temperature data were automatically recorded by an infrared probe recorder connected to the computer. In the heating stage, as shown in Figure 10c, the photo-thermal conversion curves of samples show a plateau for slow heating under illumination due to the phase-transition of PEG. The PGO-CTAB/CS6 shows a higher temperature than that of pure PEG6000 and PEG6000/CS6(6). With the increasing of PGO-CTAB, PEG melt requested more time extension and stored more thermal energy [2,40,41]. Since a short irradiation time is sufficient for the movement of low-content PGO-CTAB molecules in the lattice, the melting time of PGO-CTAB/CS6(5) was just 100.3 s. In the cooling stage, the lengths of crystallization plateaus of samples show a difference. The delayed response of melted PEG in the cooling process caused the temperature increase of these crystallization plateaus [42]. The solidification time of PCMs was consistent with their melting time, which means PCM with a long melting time also needs a long solidification time. In addition, the photothermal conversion efficiency η can also be calculated from Equation (6), and it is listed in Table 7 [43]:

(6) $$\begin{array}{c} \eta =\frac{\Delta H\times m}{Ρ\times A\times \Delta t} \end{array}$$

where m refers to the sample mass, *ΔH* is their heat enthalpy, *P* refers the light intensity, *A* is their irradiated area, and *Δt* is the time of their phase transition.

According to results in Table 7, with surfactant grafted on GO, all PGO-CTAB/CS6 show much better photothermal conversion performance than PEG6000and PEG6000/CS6(6). It is worth mentioning that the photothermal conversion efficiency of PGO-CTAB/CS6(5) reached 88.78% with good leak resistance.

**Figure 11:**
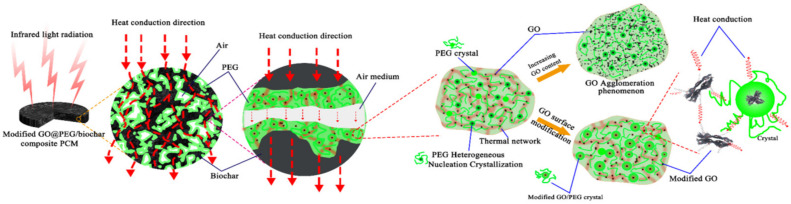
Schematic diagram of heat transfer mechanism in composite PCMs: thermally enhanced effect of GO particles, crystallization of PEG, photothermal conversion of PCMs.

Figure 11 is the schematic diagram of heat transfer mechanism in GO-PEG/biochar composite PCMs in which GO was grafted by surfactants, clearly showing the thermally enhanced effect of GO particles, the crystallization pattern of PEG, and the internal process of photothermal conversion of PCMs.

With the infrared radiation, the thermal conductivity frame built by dispersive GO particles and biochar skeleton accelerated the heat transfer in GO-PEG/biochar. Such a frame could weaken the phonon scattering, in turn, weaken its negative effect on the thermal conductivity of PCMs. Heat energy diffused throughout the PEG lattice by sympathetic vibration or phonons and then was transmitted to surroundings by conduction or radiation. GO agglomeration, crystal defects, and heterogeneity of the material, air medium in gaps would lead to thermal resistance blocking heat transmission in a composite. It was reported that it is effective to eliminate the scattering of high-frequency phonons to enhance the thermal conductivity of the material. Lowering the volume proportion of the grain boundary by lower grain boundary width or increasing grain boundary size, or improving the crystallization rate by transferring amorphous PEG to the crystalline state are practical strategies [44]. Overall, the addition of the GO-modifier and biochar effectively improved the thermal conductivity and thermal stability of PEG.

## 4. Conclusions

In this work, the relationship between the structure and properties of the GO-modifiers grafted by surfactants, the GO-PEG PCMs obtained by dispersing the above GO-modifiers in PEG6000, and the GO-PEG/biochar PCMs obtained by encapsulating above GO-PEG into biochar, was studied. CTAB, KH-560, and KH-570 grafted GO-modifiers has higher onset decomposite temperature than the pristine GO, indicating that the surfactants can improve the thermal stability of GO, and the surfactants have improved the dispersity of GO in PEG6000. The conclusions are as follows:

(1) The latent heat of PGO-CTAB, PGO-KH560, and PGO-KH570 (173.04, 186.01, 191.36 J/g, respectively) are higher than that of PGO (168.72 J/g), demonstrating the improved crystallization behavior of PEG6000 on nucleation sites contributed from surface grafted GO-modifier;

(2) The latent heat of PGO-CTAB was the highest and increased by 18.31% compared to that of PEG6000 (161.74 J/g), so GO-CTAB was considered the best thermal enhancement GO-modifier and the best candidate for the further preparation of biochar composite PCMs. The melting enthalpy was increased proportionally to PEG6000 content in the composites and ranged from 93.47 J/g to 153.52 J/g. According to the calculated value of loading efficiency E and crystallinity of PEG Fc of PGO-CTAB/CS6 PCMs, GO-CTAB has enhanced the thermal performance of the composite and helped to eliminate the confinement effects when PEG6000 was loaded on biochar in restricted volume. The induction of crystallization of GO-CTAB and biochar towards PEG6000 was also proved. PGO-CTAB/CS6(5\6) PCMs demonsteate good leak resistance.

(3) In particular, PGO-CTAB/CS6(6) is of thermal conductivity of 0.337 W/(m·K), a little higher than that of PEG6000 (0.325 W/(m·K)). With infrared radiation, The PGO-CTAB/CS6 shows a higher temperature than that of pure PEG6000 and PEG6000/CS6(6). Among them, the photothermal conversion efficiency of PGO-CTAB/CS6(6) reached 77.43% with good leak resistance. The addition of the GO-modifier and biochar effectively improved the thermal conductivity and thermal stability of PEG6000.

## Figures and Tables

**Table 1:** Latent heat performance parameters of PEG6000 and GO-PEG PCMs.

Sample	T_m_ (°C)	ΔH_m_ (J/g)	T_c_ (°C)	ΔH_c_ (J/g)
PEG6000	59.39	161.74	41.57	158.14
PGO	59.58	168.72	39.80	161.49
PGO-KH570	59.44	173.04	40.02	163.40
PGO-KH560	60.22	186.01	41.21	176.85
PGO-CTAB	60.69	191.36	40.99	181.87

**Table 2:** Latent heat performance parameters of various PCMs.

Sample	Tm (°C)	ΔH_m_ (J/g)	Tc (°C)	ΔHc (J/g)
PEG6000	59.39	161.74	41.57	158.14
PEG6000/CS6(6)	58.62	99.32	41.10	93.91
PGO-CTAB/CS6(5)	60.83	93.47	40.02	89.97
PGO-CTAB/CS6(6)	61.05	106.51	41.20	102.70
PGO-CTAB/CS6(7)	60.42	132.10	40.95	127.05
PGO-CTAB/CS6(8)	60.56	153.52	41.89	147.83

**Table 3:** Loading efficiency E and the crystallinity of PEG Fc of various PCMs.

Sample	PEG wt%	E	Fc
PEG6000	100%	100%	100%
PGO	99.4%	103.2%	98.96%
PGO-KH570	99.4%	105.2%	98.31%
PGO-KH560	99.4%	113.4%	98.64%
PGO-CTAB	99.4%	116.7%	98.62%
PEG6000/CS6(6)	60%	60.4%	98.37%
PGO-CTAB/CS6(5)	50%	57.3%	99.23%
PGO-CTAB/CS6(6)	60%	65.4%	99.32%
PGO-CTAB/CS6(7)	70%	81.0%	99.19%
PGO-CTAB/CS6(8)	80%	94.2%	99.25%

**Table 4:** Average crystal size of pure PEG6000 and various composite PCMs.

Sample	Crystal Size (nm)
Pure PEG6000	12.04
PGO	11.83
PEG6000/CS6(6)	11.63
PGO-KH570	11.30
PGO-KH560	11.68
PGO-CTAB	11.64
PGO-CTAB/CS6(5)	11.22
PGO-CTAB/CS6(6)	11.17
PGO-CTAB/CS6(7)	11.24
PGO-CTAB/CS6(8)	11.54

**Table 5:** Leakage stability parameters of pure PEG6000 and various composite PCMs.

Sample	Mass (g)	Diameter (mm)	Leakage Area (mm^2^)	Leakage Rate Φ (%)
Pure PEG	0.235	12.7	579.42	100.00
PGO-KH560	0.236	12.7	447.05	77.15
PGO-KH570	0.230	12.7	835.66	144.22
PGO-CTAB	0.255	12.7	894.28	154.34
PGO-CTAB/CS6(5)	0.246	12.7	0.00	0.00
PGO-CTAB/CS6(6)	0.242	12.7	99.15	17.11
PGO-CTAB/CS6(7)	0.235	12.7	353.70	61.04
PGO-CTAB/CS6(8)	0.239	12.7	543.91	93.87

**Table 6:** Thermal conductivity of pure PEG6000 and various composite PCMs.

Sample	Thermal Conductivity Value K (W/m·K)
PEG6000	0.325
PEG6000/CS6(6)	0.299
PGO	0.628
PGO-KH570	0.398
PGO-KH560	0.424
PGO-CTAB	0.450
PGO-CTAB/CS6(5)	0.291
PGO-CTAB/CS6(6)	0.337
PGO-CTAB/CS6(7)	0.293
PGO-CTAB/CS6(8)	0.284

**Table 7:** Photo-thermal conversion performance parameters of phase change composites.

Sample	m/(g)	∆Hm/(J/g)	P/(mW/cm^2^)	∆t/s	η/%
PEG6000	0.2309	161.74	200	633.0	44.63
PEG6000/CS6(6)	0.2540	86.57	200	543.0	56.36
PGO-CTAB/CS6(5)	0.2414	93.47	200	100.3	88.78
PGO-CTAB/CS6(6)	0.2428	106.51	200	131.8	77.43
PGO-CTAB/CS6(7)	0.2474	132.10	200	144.6	89.19
PGO-CTAB/CS6(8)	0.2503	153.53	200	176.0	86.17

## Data Availability

The data presented in this study are available on request from the corresponding author.
